# pH-Assisted multichannel heat shock monitoring in the endoplasmic reticulum with a pyridinium fluorophore†

**DOI:** 10.1039/d4sc01977f

**Published:** 2024-06-04

**Authors:** Sandip Chakraborty, Anivind Kaur Bindra, Anagha Thomas, Yanli Zhao, Ayyappanpillai Ajayaghosh

**Affiliations:** a CSIR-National Institute for Interdisciplinary Science and Technology (CSIR-NIIST) Thiruvananthapuram 695 019 India ajayaghosh@niist.res.in; b Academy of Scientific and Innovative Research (AcSIR) Ghaziabad 201002 India; c Department of Chemistry, SRM Institute of Science and Technology Chennai 603203 India ajayagha@srmist.edu.in; d School of Chemistry, Chemical Engineering and Biotechnology, Nanyang Technological University 21 Nanyang Link 637371 Singapore zhaoyanli@ntu.edu.sg

## Abstract

Heat shock is a global health concern as it causes permanent damage to living cells and has a relatively high mortality rate. Therefore, diagnostic tools that facilitate a better understanding of heat shock damage and the defense mechanism at the sub-cellular level are of great importance. In this report, we have demonstrated the use of a pyridinium-based fluorescent molecule, PM-ER-OH, as a ‘multichannel’ imaging probe to monitor the pH change associated with a heat shock in the endoplasmic reticulum. Among the three pyridinium derivatives synthesized, PM-ER-OH was chosen for study due to its excellent biocompatibility, good localization in the endoplasmic reticulum, and intracellular pH response signaled by a yellow fluorescence (*λ*_max_ = 556 nm) at acidic pH and a far red fluorescence (*λ*_max_ = 660 nm) at basic pH. By changing the excitation wavelength, we could modulate the fluorescence signal in ‘turn-ON’, single excitation ratiometric and ‘turn-OFF’ modes, making the fluorophore a ‘multichannel’ probe for both *ex vitro* and *in vitro* pH monitoring in the endoplasmic reticulum. The probe could efficiently monitor the pH change when heat shock was applied to cells either directly or in a pre-heated manner, which gives insight on cellular acidification caused by heat stress.

## Abstract 2

An endoplasmic reticulum-targeting fluorescent pH sensor can be used in “turn-on”, “ratiometric” and “turn-off” modes in the same pH range by changing the excitation wavelength of the microscope to monitor heat shock in cells.

## Graphical abstract

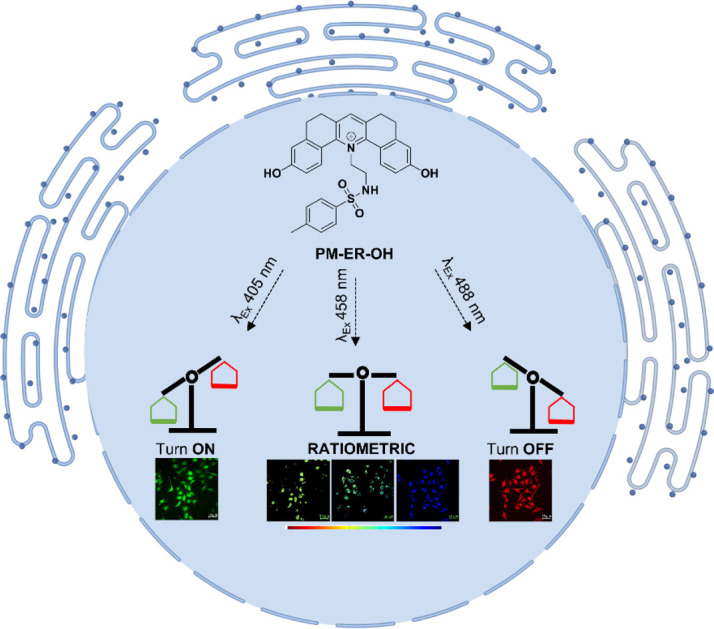

## Introduction

Heat shock is a major health concern due to the occurrence of heat waves and fire hazards, and is a major life threat leading to human mortality across the globe.^1^ An increase of 1.4–5.8 °C in global temperature is expected to severely impact on the environment, particularly human health, by the end of this century.^2^ A rise in body temperature above 40.6 °C can spontaneously cause serious injury to the tissues and the nervous system, leading to multiple organ failure and death.^3^ Though a long-known health concern, the mechanistic aspects behind the pathological changes that occur during heat shock are rather poorly understood.^4^ Therefore, elucidating the damage mechanism at the sub-cellular level due to “heat shock” may offer a better treatment modality and be lifesaving.

Fluorescence is a strong and sensitive tool for the probing and imaging of sub-cellular changes.^5^ Characterized by a favourable combination of fast response, non-invasive nature, good spatiotemporal resolution and advanced instrumental evolution, fluorescence-based imaging has assumed a pivotal role in clinical diagnosis.^6^ The specific organelle targeting ability of small molecular fluorescent probes is preferred over various nanoparticles and fluorescent proteins.^7^

Recently, fluorescent probes were used to quantitatively monitor the pH, sulphur dioxide and glutathione levels within the lysosomes and mitochondria during heat shock incidents.^8^ pH is one of the most important analytes in bioimaging, as the hydronium ion plays a crucial role in maintaining cellular homeostasis.^9^ Although there have been several ways of measuring cytosolic pH for a long time, improved techniques and tools for accurately monitoring the pH of various organelles have opened new doors of information in recent years. The distinct function of each organelle has been found to be highly pH dependent. Cellular functions such as apoptosis, autophagy, enzymatic degradation, protein folding, transportation processes from one organelle to other *etc.* have been found to be closely dependent on the pH of the surroundings.^10^ To date, heat shock monitoring at the sub-cellular levels has been achieved mostly using lysosome targeting pH probes. However, hyperthermia has been found to initially affect the endoplasmic reticulum (ER) and Golgi network, followed by the mitochondria and later, the lysosomes.^11^

Since the ER plays a crucial role in protein and lipid synthesis, protein folding and calcium homeostasis, maintaining a consistent pH is essential for sustaining physiological functions.^12^ Furthermore, several heat shock proteins (HSP) in the ER are known to shield cells from heat damage.^13^ Thus, developing fluorescent probes capable of explicitly monitoring pH changes within the ER during heat shock can offer unique insights that may further advance the diagnosis and allied treatment modalities.^14^ There are several reports of ratiometric pH probes in the literature for acidic, neutral, and basic pH ranges that can be used for various organelles, depending on the range of pH sensitivity.^15^ However, most of them have a narrow sensitivity range, which is a major concern where there is a possibility of large pH variations.

To overcome this problem, a few strategies have been adopted, such as covalently attaching two pH sensors of different p*K*_a_ and incorporating multiple fluorescent pH sensors into a polymer matrix. However, these strategies suffer from difficult synthesis and inconsistency in the ratio of fluorophores in different batches of the polymer matrix. To overcome these limitations and to extend the pH sensitivity range, introducing two p*K*_a_s could be a great solution. In addition, good aqueous solubility, large stokes shift, visible to NIR range emission, ratiometric response and good photostability can be great additions for a useful pH probe.^16–19^ We herein report a pyridinium-based pH sensor, PM-ER-OH, which shows a broad range of pH sensitivity due to it having two p*K*_a_s and emits in the yellow to far red region in a ratiometric manner based on pH variation. It shows excellent photostability, biocompatibility and no alteration in fluorescence response with increase in temperature due to its rigid structure, making it an excellent candidate for monitoring the intracellular pH change caused by heat shock.

## Results and discussion

**Scheme 1:**
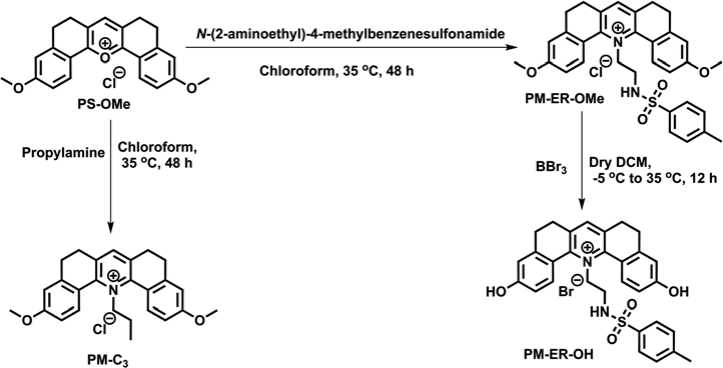
Synthesis of PM-C_3_, PM-ER-OMe and PM-ER-OH, starting from our previously reported molecule PS-OMe.

Our previous experience with pyrylium fluorophores helped us in the design of three new pyridinium fluorophores, PM-C3, PM-ER-OMe and PM-ER-OH.^20^ During the design of these molecules, we set our target as: (i) introducing an organelle directing group, (ii) developing a ratiometric fluorophore having an unaffected signal at elevated temperature, and (iii) monitoring intracellular pH change during heat shock. Syntheses of the probes were achieved by converting the pyrylium fluorophore to the corresponding pyridinium derivative, as shown in Scheme 1.^21^ The pyrylium dye PS-OMe was treated with propylamine and *N*-(2-aminoethyl)-4-methylbenzenesulfonamide to obtain the pyridinium derivatives of PM-C3 and PM-ER-OMe, respectively, in moderate yields. PM-ER-OH was synthesized by demethylation of PM-ER-OMe using boron tribromide (86% yield). The procedure and characterization data are provided in the ESI.†

Out of the three pyridinium molecules, PM-C3 does not have a specific targeting group for the ER, PM-ER-OMe can target the ER but cannot respond to pH, whereas PM-ER-OH features an ER targeting moiety and two hydroxy groups that quantitatively respond to changes in pH. The absorption and emission spectra of the three compounds were first recorded in chloroform at a concentration of 10 μM (Fig. S2†). PM-C3 showed an absorption maximum at 430 nm, with an extinction coefficient (*ε*) of 3.9 × 10^4^ M^−1^ cm^−1^. For PM-ER-OMe, the absorption maximum was slightly red-shifted to 436 nm and the extinction coefficient (*ε*) was 2.69 × 10^4^ M^−1^ cm^−1^. Both the molecules exhibited similar emission properties with a maximum at 566 nm. However, the absorption and the emission of PM-ER-OH exhibited a significant red-shift with maxima at 445 nm and 585 nm, respectively. The fluorescence quantum yields of the molecules were measured in EtOH, using Coumarin 153 as the standard (*Φ*_f_ = 0.38 in EtOH). In ethanol, the quantum yield of PM-C3 was 0.54 and for PM-ER-OMe, it was 0.62. PM-ER-OH showed a slightly lower quantum yield (0.57) than PM-ER-OMe (0.62), probably caused by the decrease in the donor strength. However, the quantum yield was significantly higher than that of PS-OH, which could be due to the enhanced delocalization of the less electronegative acceptor nitrogen atom and better intramolecular charge transfer.

The pH response of the three molecules was first evaluated in freshly prepared PBS buffer (10 mM, pH 4, 7.4, and 10). As expected, no significant change in absorbance or emission was observed for a solution of PM-C3. However, in the case of PM-ER-OMe, increasing pH led to a slight decrease in the intensity of absorption and emission (Fig. S3†), probably due to a partial photoinduced electron transfer (PET) of the sulfonamide moiety that is in close proximity to the acceptor part of the PM-ER-OMe.

**Fig. 1:**
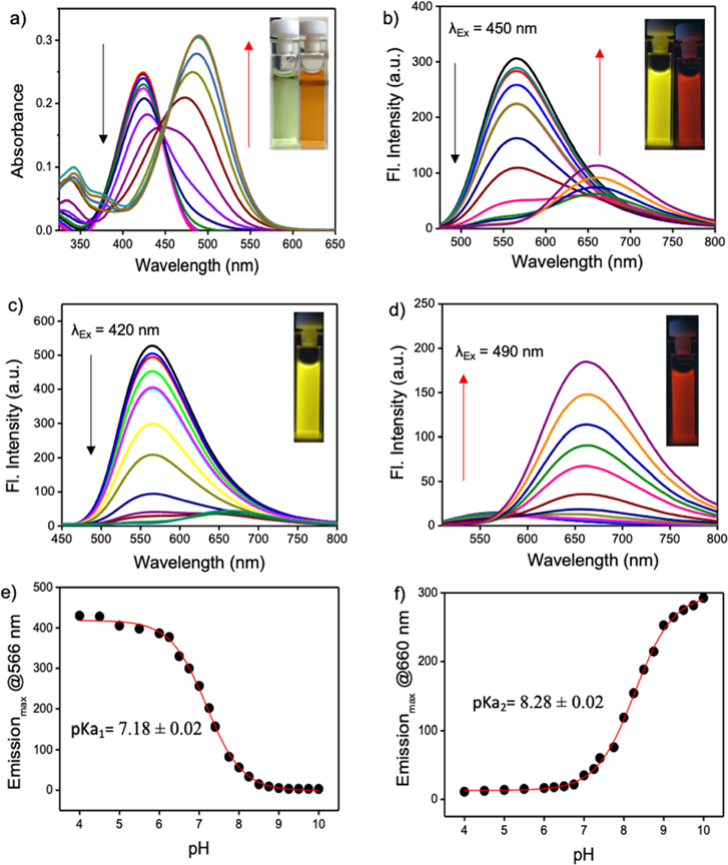
(a) Variable pH absorption spectra of PM-ER-OH (10 μM) in PBS buffer starting from pH 4 to pH 10. In the inset, photographs of the probe at pH 4 (yellow) and 10 (orange) in daylight. (b) Ratiometric emission spectra of PM-ER-OH when excited at 450 nm. In the inset, photographs of the probe at pH 4 (yellow) and 10 (red) under 365 nm light. (c) Turn OFF emission spectra of PM-ER-OH when excited at 420 nm. Representative image of the probe in pH 4 buffer under 365 nm light. (d) Turn ON emission spectra at 490 nm excitation. Representative image of the probe in pH 10 buffer under 365 nm light in the inset. (e) Secondary plot derived from (c) and p*K*_a1_ determination. (f) p*K*_a_2__ calculation of PM-ER-OH using the secondary plot constructed from (d). The corresponding photographs of the buffered solutions of the probe are shown in the insets.

The pH response of PM-ER-OH was recorded by the change in the absorption maximum (*λ*_max_) of PM-ER-OH in acidic (pH 4) and alkaline (pH 10) solutions. The absorption maxima were found to be at 420 and 490 nm, respectively, with a near isosbestic point at 450 nm (Fig. 1a), that is indicative of an operational equilibrium between the protonated and deprotonated species.

Nevertheless, not having a clear isosbestic point in the absorbance spectra indicated that the transition from the protonated state to the completely deprotonated state is not restricted between two forms of the molecule and the deprotonation of PM-ER-OH progresses through multiple steps. The emission maximum at 566 nm gradually decreased with increasing pH (pH 4 to 10), with a concomitant increase of a new peak appearing at *λ*_max_ = 660 nm upon excitation at the isosbestic wavelength (*λ*_ex_ = 450 nm), resulting in a ratiometric fluorescence response (Fig. 1d). Thus, this probe can act as a single excitation ratiometric probe. When excited at the first absorbance maximum (*λ*_ex_ = 420 nm), the emission at 566 nm gradually decreased with increasing pH, with no detectable emission at 660 nm (Fig. 1b), thereby affording a turn-OFF response at 566 nm. Similarly, when excited at the second absorbance maximum (*λ*_ex_ = 490 nm), the emission at 660 nm steadily increased, with barely any emission at 566 nm (Fig. 1e), confirming a turn-ON response at 660 nm in the same pH range. Thus, the probe PM-ER-OH serves as an excitation wavelength dependent ‘ratiometric’, ‘turn-ON’ or ‘turn-OFF’ probe without any significant spectral overlap and high Stokes shifts (pH 4: 6142 cm^−1^, pH 10: 5257 cm^−1^). A non-linear Boltzmann fitting of the secondary plots generated from the emission spectra resulted in calculated p*K*_a_1__ and p*K*_a_2__ values of 7.18 ± 0.02 (Fig. 1c) and 8.28 ± 0.02 (Fig. 1f), respectively. These observations are indicative of a two-step deprotonation process involving the terminal –OH groups, both of which are well in the range of the physiologically relevant pH window. These results corroborate the wide range of pH monitoring capability of PM-ER-OH inclusive of the normal ER pH (7.2) and monitoring the heat shock induced damage mechanism of the ER *via* alteration in its pH.

The fluorescence quantum yield of PM-ER-OH in PBS buffer solution of pH 4 and 10 was found to be 0.12 and 0.04, respectively. The pH dependent emission properties of PM-ER-OH were recorded using 405, 458 and 488 nm excitation wavelengths as these are readily available in most confocal laser scanning microscopes (CLSMs) (Fig. S4†).

**Fig. 2:**
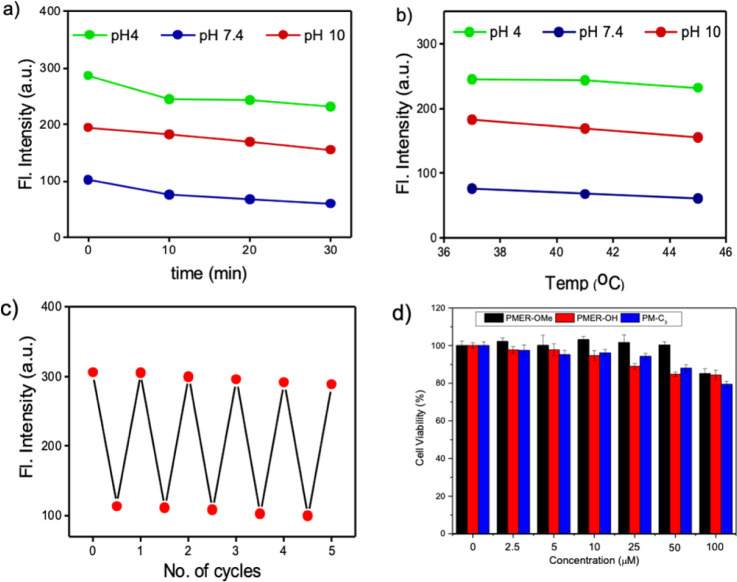
(a) Plot showing the photostability of the probe PM-ER-OH at pH 4, 7.4 and 10. The emission maximum at the corresponding pH is plotted against time. (b) Influence of temperature on the fluorescence behaviour of PM-ER-OH at acidic, neutral, and basic pH. (c) Reversibility of PM-ER-OH (10 μM) towards acid (pH 4) and base (pH 10). (d) Cytotoxicity evaluation of PM-ER-OMe, PM-ER-OH and PM-C3 when incubated with HeLa cells for 24 h *via* an MTT assay.

Excitation with 405 nm and 488 nm wavelengths resulted in typical pH dependent “ON/OFF” emission profiles, and excitation using a 458 nm laser source showed a single excitation “ratiometric” pH response, without any significant spectral overlap due to the unprecedentedly high Stokes shift of the PM-ER-OH. The photostability of the fluorophore is confirmed by exposing a 10 μM solution of PM-ER-OH in PBS buffer at different pH (pH 4, 7.4 and 10) to a Hg lamp (200 W, 365 nm long pass filter) for 30 minutes. The plot of emission intensity (*λ*_max_ = 566 nm for pH 4 and 7.4, and *λ*_max_ = 660 nm for pH 10) *vs.* time showed >80% fluorescence retention after 30 minutes of irradiation, which is indicative of the superior photostability of PM-ER-OH (Fig. 2a). The propensity towards free rotation induced non-radiative transitions in the extended conjugated fluorophores with large Stokes shifts, making their fluorescence response susceptible to changes with temperature.^22^ Therefore, we investigated the temperature-dependent emission of PM-ER-OH at 35, 41, and 45 °C in acidic (pH 4), neutral (pH 7.4), and alkaline (pH 10) PBS buffer. To our satisfaction, the emission profiles in terms of wavelength and intensity of the rigid pentacyclic pyridinium fluorophore (Fig. 2b) were found to be unaffected by variations in temperature due to the lack of structural flexibility for non-radiative pathways. Multiple excitations up to five cycles at pH 4 and 10 did not show any significant changes in emission intensity, confirming the probe's competence in monitoring dynamic changes in pH (Fig. 2c). Selectivity experiments involving analytes such as metal ions, anions, reactive oxygen species, bio-thiols, *etc.* at three different pH levels (4, 7.4 and 10, Fig. S5a–c†) confirmed that PM-ER-OH is responsive towards H^+^ or OH^−^ only. Cytotoxicity is a major concern for organic fluorophores to be used as imaging agents. Therefore, we checked the biocompatibility of all three derivatives under study (*c* = up to 100 μM) on HeLa cells using a 3-[4,5-dimethylthiazol-2-yl]-2,5 diphenyl tetrazolium bromide (MTT) assay (up to 24 h), which showed low cytotoxicity and cell viability of ∼85% (Fig. 2d).

Encouraged by the photophysical superiority and excellent biocompatibility of PM-ER-OH, we went ahead and tested its biological application for imaging pH variations at the sub-cellular level using HeLa cells that were obtained from the American Type Culture Collection (ATCC, Manassas, VA, USA. See ESI† for details). Initially, the cellular uptake of the probes was checked, and they were found to be internalized by the cells in less than 10 min. So, we decided to perform all imaging experiments after 10 min of dye treatment. Colocalization studies were conducted using PM-ER-OH (Fig. S6a†) and the control molecule PM-C3 (Fig. S6c†), and were compared to that using the commercially available ER-tracker blue. The Pearson's coefficients were found to be 0.77 (Fig. S6b†), and 0.50 (Fig. S6d†) for PM-ER-OH, and PM-C3, respectively. This result suggests moderately efficient colocalization of the probe PM-ER-OH in the ER and corroborates the vital role of the targeting groups for localizing the probe at the sub-cellular level. The *in vitro* photostability of PM-ER-OH was found to be superior to that of the commercially available ER-tracker blue (Fig. S7†). Intracellular pH imaging of HeLa cells after incubating for 10 min was performed using the confocal fluorescence imaging technique, equipped with laser excitation sources of 405, 458, and 488 nm. The images were captured in the green (500–550 nm) and red channels (625–750 nm). Intracellular pH was clamped at 4.2, 7.4, and 9.2 using nigericin in the presence of an extracellular buffer prior to the experiments following the reported protocols. Akin to the fluorescence changes in solution, excitation using a 405 nm laser source resulted in a gradual decrease in the emission intensity with increasing pH when monitored in the green channel. The observed fluorescence intensity followed the order: pH 4.2 (intensity = 32.96 a.u.) > pH 7.4 (intensity = 24.25 a.u.) > pH 9.2 (intensity = 12.90 a.u.), with almost no detectable fluorescence in the red channel.

**Fig. 3:**
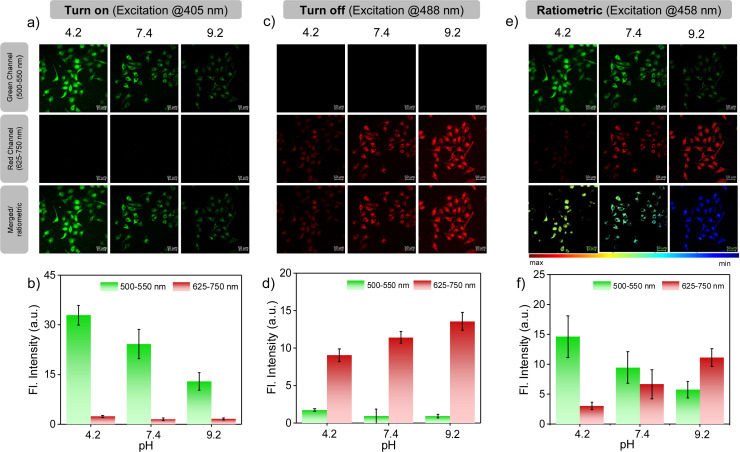
CLSM images of HeLa cells after 10 minutes of incubation with the probe PM-ER-OH (10 μM) in buffer solution of different pH in the presence of 10 μM nigericin. Images in the green and red channels along with merged or ratiometric images, upon excitation with (a) 405 nm laser, (c) 488 nm laser, and (e) 458 nm laser. The bar diagrams represent the values of fluorescence intensities (in a.u.) obtained from the image analysis of (b) 3a, (d) 3c and (f) 3e. The bar denotes the ratio of the green channel over the red channel with pH variation from 4.2 (max) to 9.2 (min). The colour of the bars represents the corresponding channel. Scale bar 50 μm.

This observation confirms a “turn-ON” fluorescence response during the acidification of cells upon excitation at 405 nm (Fig. 3a). On the other hand, the fluorescence intensity in the red channel was found to increase with increasing pH (intensity = 9.05 a.u. at pH 4.2, 11.42 a.u. at pH 7.5 and 13.58 a.u. at pH 9.2) upon excitation at 488 nm, with no simultaneous emission change in the green channel (Fig. 3c and d), thus corroborating the ‘turn-OFF” response of PM-ER-OH during the cellular acidification process. Use of 458 nm laser excitation with increasing intracellular pH resulted in a decrease in the fluorescence intensity in the green channel (intensity = 14.65 a.u. at pH 4.2, 9.47 a.u. at pH 7.5 and 5.75 a.u. at pH 9.2), with a concomitant increase in the emission intensity in the red channel (intensity = 3.01 a.u. at pH 4.2, 6.66 a.u. at 7.5, and 11.13 a.u. at pH 9.2) (Fig. 3e and f), leading to a typical single excitation ratiometric response. These results strongly support the fact that the probe is efficient for bioimaging, particularly for monitoring intracellular pH changes.

Heat induced cellular damage is irreversible, even if the cells are later cooled to normal temperature.^23^ However, the damage due to heat shock can be significantly curtailed by a process called hormesis, wherein cells are preconditioned at mild heat for a short period of time.^24^ Since pH is one among the factual parameters accepted universally for understanding cellular homeostasis, lysosomal pH probes were recently reported for monitoring heat shock. However, the endoplasmic reticulum is impacted prior to lysosomes by any heat shock event and the activation of several heat shock proteins located in the ER helps to prevent the heat damage that preserves homeostasis during hormesis. Therefore, monitoring pH variations in the ER during heat shock can offer insights into cellular damage pathways at an early stage when compared to lysosomal pH probes. Furthermore, we believe that ER targeting PM-ER-OH can be an ideal candidate for monitoring heat shock events at the sub-cellular level. Two sets of experiments were designed for heat shock mapping in live HeLa cells. (a) The cells were heated directly to 41 and 45 °C from the normal cell temperature (37 °C), and (b) the cells were pre-heated for 3 h, just below the prescribed heat shock temperature, *i.e.*, at 40 °C and then heated to 41 and 45 °C. The fluorescence images of both sets at 41 and 45 °C were evaluated after exciting with three laser sources and at two emission channels using the probe PM-ER-OH as described earlier and the intensity change was calculated with respect to the intensity at 37 °C, which was the same for both sets of cells.

**Fig. 4:**
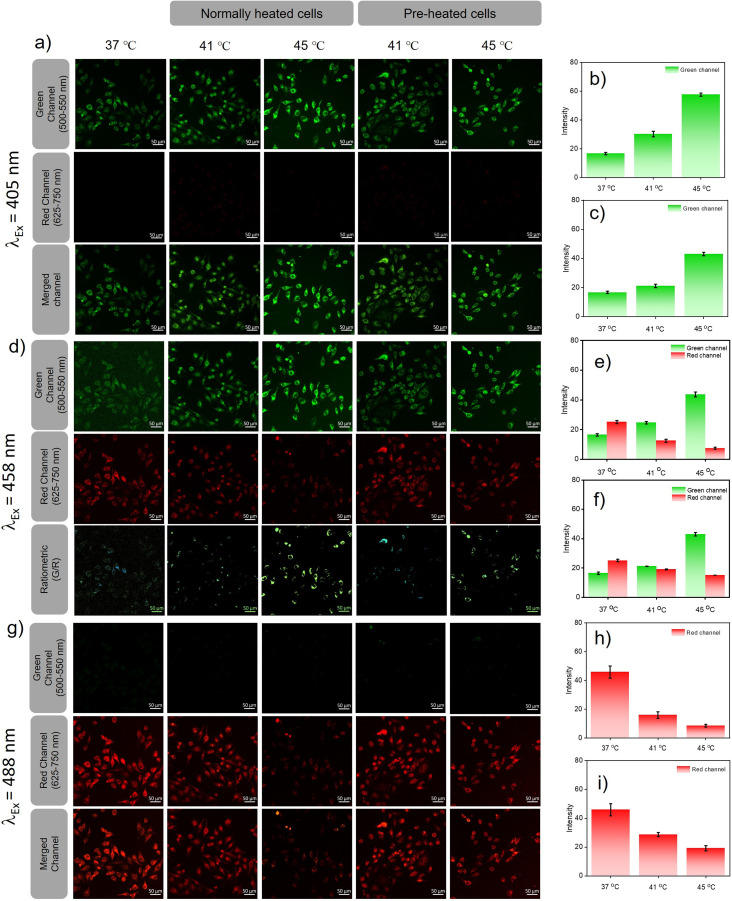
*In vitro* heat shock mapping *via* CLSM imaging of HeLa cells incubated with PM-ER-OH (10 μM), after inducing heat shock in two different ways. (a) Images of heat-treated cells in green, red, and merged channels using 405 nm laser excitation. (b) and (c) The fluorescence intensity increment in the green channel for the directly heated and the pre-heated cells, respectively. (d) green and red channel images along with the constructed ratiometric images (green/red) upon excitation at 458 nm. (e) and (f) The fluorescence intensity change in the green and red channel for both directly heated and pre-heated cells, respectively. (g) Fluorescence images in the green and red channel upon excitation at 488 nm. (h) and (i) Fluorescence intensity decrease in the red channel for the directly heated and the pre-heated cells, respectively. Scale bar: 50 μm.

Excitation at 405 nm resulted in an increase in the fluorescence intensity in the green channel (Fig. 4a) with an increase in temperature (intensity = 1.8-fold at 41 °C and 3.46-fold at 45 °C w.r.t the intensity at 37 °C) for directly heated cells (Fig. 4b) and (intensity = 1.2-fold at 41 °C and 2.58-fold at 45 °C w.r.t 37 °C) for pre-heated cells (Fig. 4c), with no detectable emission in the red channel. For 458 nm laser excitation (Fig. 4d), a similar result was observed when monitored in the green channel (intensity = 1.5-fold at 41 °C, and 2.3-fold at 45 °C over the intensity at 37 °C) for directly heated cells and (1.18-fold at 41 °C, and 1.45-fold over the intensity at 37 °C) at 45 °C for pre-heated cells. For directly heated cells, the temperature dependent increase in fluorescence intensity was higher than that for the pre-heated cells. Cellular acidification is therefore correlated directly to the increase of temperature. On the contrary, when excited at 458 nm, the fluorescence intensity in the red channel decreased with increasing temperature for both directly heated (intensity = 0.5-fold that at 41 °C and 0.3-fold that 45 °C over the intensity at 37 °C) and pre-heated (intensity = 0.76-fold at 41 °C and 0.6-fold at 45 °C w.r.t the intensity at 37 °C) cells (Fig. 4e and f). If we take a ratio of intensities (green/red), it showed a higher increment for directly heated cells (intensity ratio = 3.01-fold at 41 °C and 7.64-fold at 45 °C), than pre-heated cells (intensity ratio = 1.56-fold at 41 °C and 2.43-fold at 45 °C) cells. Images taken in the red channel using a 488 nm laser as the excitation source (Fig. 4g) showed a similar trend for both directly heated (intensity = 0.35-fold at 41 °C, and 0.19-fold at 45 °C over the intensity at 37 °C) (Fig. 4h) and pre-heated cells (intensity = 0.63-fold at 41 °C and 0.42-fold at 45 °C) (Fig. 4i). Though similar trends in fluorescence variations with increasing temperature were observed in the green channel upon excitation at 405 or 458 nm, the intensity increments for the directly heated cells were significantly higher than that of the pre-heated cells. However, the decrease in fluorescence intensity was less predominant for the pre-heated cells when compared to that for the directly heated cells under 458 and 488 nm excitation. These observations suggest that conditioning of the cells at an elevated temperature (40 °C) below hyperthermia could resist cellular acidification far better than direct exposure of cells to hypothermic conditions. Our results confirm that heat shock-induced dysfunction of the ER can be directly monitored through the corresponding changes in pH. Pre-heating the cells below hyperthermic conditions signals the production and activation of HSPs, and as a result, the cells were protected even when the temperature rose above the tolerance level. The acidification of the ER reduces the pH of the cell, which most likely stimulates an increase in lysosomal pH *via* a neutralization process as previously reported.

## Conclusions

In conclusion, by virtue of the large Stokes shift and excitation dependent emission, the newly developed pentacyclic pyridinium fluorophore PM-ER-OH functions as a ‘multi-channel’ active fluorescent probe for the *in vitro* imaging of pH variations associated with heat shock in live cells. The lack of backbone flexibility and the presence of a sub-cellular targeting moiety along with intrinsic silence towards hyperthermic conditions facilitates live imaging of heat shock-induced dysfunction mechanisms in the endoplasmic reticulum. Moreover, the favorable combination of biocompatibility, physiological solubility, photo and chemical stability, and reasonable ER colocalization of PM-ER-OH allows for reliable monitoring of pH, both exogenously and endogenously. PM-ER-OH is probably the first fluorescent probe reported for heat shock pH mapping in the endoplasmic reticulum. Our study confirms that cellular exposure to a sudden hyperthermic wave can be more fatal than a slow rise in temperature. Our findings related to variations in pH within the endoplasmic reticulum during heat shock are useful in elucidating the role of heat shock protein activation during hyperthermia, leading to a better understanding of the mechanistic pathways behind heat-induced cellular damage and related pathologies, thereby leading to a better treatment protocol and efficacy.

## Data availability

All the data that supports this article have been included in the main text and ESI† of the manuscript.

## Author contributions

S. Chakraborty: conceptualization, synthesis, characterization, spectroscopic experiments and analysis, writing and revision of the manuscript. A. K. Bindra: cell culture, MTT assay, imaging and image analysis, writing and revision of the manuscript. A. Thomas: synthesis and characterization. Y. Zhao: supervision of imaging experiments, manuscript writing, review and editing, funding. A. Ajayaghosh: conceptualization, project supervision and administration, manuscript writing, review and editing, funding.

## Conflicts of interest

There are no conflicts to declare.

## Supplementary Material

SC-015-D4SC01977F-s001
