# Supplementary material for: pH-Assisted multichannel heat shock monitoring in the endoplasmic reticulum with a pyridinium fluorophore
Source: Chem Sci. 2024 Jun 4;15(28):10851–7. doi: 10.1039/d4sc01977f (PMC11253182; doi:10.1039/d4sc01977f)
Supplement: SC-015-D4SC01977F-s001 [file SC-015-D4SC01977F-s001.pdf]

Electronic Supplementary Material (ESI) for Chemical Science.
This journal is © The Royal Society of Chemistry 2024

pH-Assisted Multichannel Heat Shock Monitoring in Endoplasmic Reticulum with a Pyridinium Fluorophore

Sandip Chakraborty [a,b,⊥], Anivind Kaur Bindra [d,⊥], Anagha Thomas [a,b], Yanli Zhao [d,*], Ayyappanpillai Ajayaghosh [a,b,c,*]

[a] Chemical Sciences and Technology Division, CSIR-National Institute for Interdisciplinary Science and Technology (CSIR-NIIST), Thiruvananthapuram 695019, India. E-mail: ajayaghosh@niist.res.in

[b] Academy of Scientific and Innovative Research (AcSIR), CSIR- Human Resource Development Centre, Ghaziabad 201002, India.

[c] Department of Chemistry, SRM Institute of Science and Technology, Chennai 603203, India. E-mail: ajayagha@srmist.edu.in

[d] School of Chemistry, Chemical Engineering and Biotechnology, Nanyang Technology University,21 Nanyang Link, Singapore 637371, Email: zhaoyanli@ntu.edu.sg

Supporting Information

Sl. No.		Page No.
1	Materials and methods	S3
	1.1 Synthesis and characterization	S3-S6
2	Optical measurements	S6
	2.1 Basic photophysical data of the molecules	S6
	2.2 PM-C_3 and PM-ER-OMe pH response	S7
	2.3 Fluorescence Quantum Yield measurement	S7
3	Laser wavelength compatibility	S8
4	Selectivity of PM-ER-OH	S8
5	General Strategies for cellular studies	S8
	5.1 Cell culture	S8
	5.2 MTT assay	S9
	5.3 Co-localization	S9
	5.4 Photobleaching/photostability Studies	S10
	5.5 pH variation	S10
	5.6 Heat shock imaging	S11
9	^{1}H NMR, ^{13}C NMR and HRMS of **PM-C_3**, **PM-ER-OMe** and **PM-ER-OH**	S11- S15

1. Materials and Methods:

All reagents and dry solvents were purchased from commercial sources and were used as such. Column chromatography was done using silica gel 100-200 mesh. ^{1}H and ^{13}C NMR were recorded on a Bruker Advance II spectrometer at 500 MHz and 125 MHz, respectively with solvents mentioned with the NMR data. Data are reported as follows: chemical shift in ppm (δ), multiplicity (s = singlet, d = doublet, t = triplet, q = quartet, coupling constant (Hz) and integration. HRMS analysis was recorded on a Thermo Scientific Exactive LCMS instrument by electrospray ionization method with ions given in m/z using Orbitrap analyzer.

Electronic absorption spectra were recorded in a Shimadzu UV–2600 spectrophotometer. All emission spectra were taken in Horiba Fluorolog ─ 3 Jovin Yoon. The cell imaging experiments were carried out by a Zeiss LSM710 Airyscan CLSM (confocal laser scanning microscope). Optical density (OD) for the cellular experiments was recorded using the microplate reader (infinite M200, TECAN).

1.1 Synthesis and Characterization

Synthesis of PM-C$_3$, PM-ER-OMe and PM-ER-OH

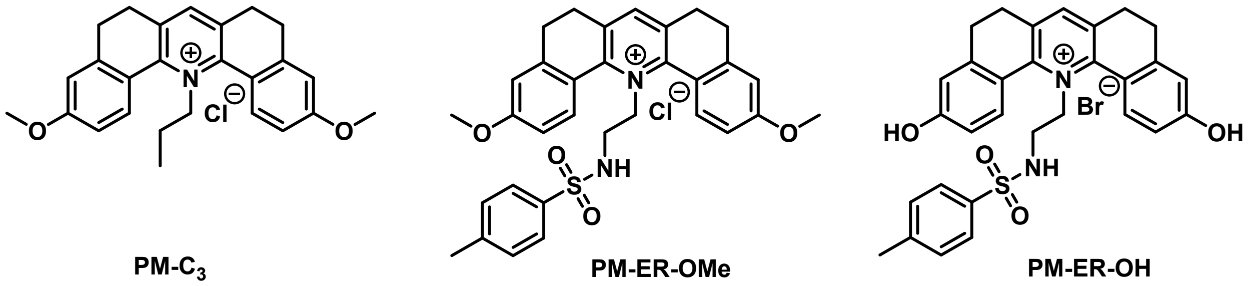

Fig S1: Structures of the pentacyclic pyridinium molecules used in this work.

Synthesis of PM-C$_3$:

PS-OMe (0.5 g, 1.26 mmol) was dissolved in 200 mL of chloroform and kept with stirring in a round bottom flask. Then Propylamine (0.359 g, 6.08 mmol) was added to it dropwise to the chloroform solution. The reaction mixture was kept with stirring at 35 °C for 4 h. Completion of the reaction was confirmed by checking TLC. Excess amine was removed from the reaction mixture by washing with water (3 x 100 mL). Organic layer was collected, dried over sodium sulphate and

solvent was removed under vacuum. The compound was redissolved using minimum volume of methanol and excess of cold diethyl ether was added to it for getting compound precipitation. **PM-C_3** (0.38 g, 71.5%) was collected by filtration and dried in oven at 50 °C to afford yellow powder. It was characterized by ^1H, ^{13}C NMR and HRMS.

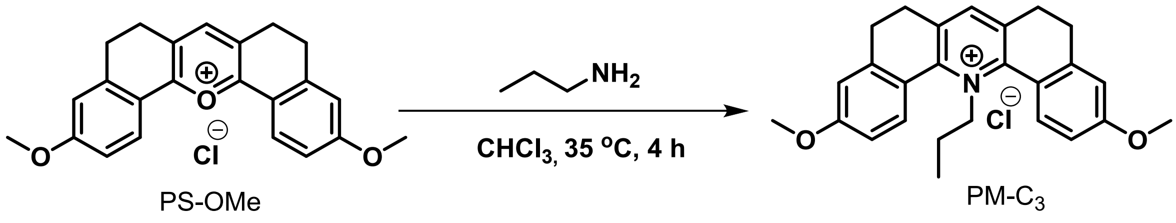

Yield: 71.5 %

^1H NMR (500 MHz, $CDCl_3$): 8.28 (d, J = 5 Hz, 2H), 7.99 (s, 1H), 7.14-7.12 (d, J = 5 Hz, 2H), 6.90 (s, 2H), 5.24-5.22 (t, J = 5 Hz, 2H), 3.91 (s, 6H), 3.05-3.04 (d, J = 2.5 Hz, 2H), 2.9-2.89 (d, J = 2.5 Hz, 4H), 2.75-2.74 (d, J = 2.5 Hz, 2H), 1.31-1.27 (m, 2H), 0.37-0.34 (t, J = 7.5 Hz, 3H)

^{13}C NMR (125 MHz, $CDCl_3$): 162.9, 153.3, 142.9, 136.3, 136.35, 120.9, 114.7, 113.5, 65.9, 55.8, 28.6, 28.2, 23.2, 10.2

HRMS: Calculated mass = 386.2115, Experimental mass = 386.2134

Synthesis of PM-ER-OMe:

PS-OMe (0.5 g, 1.26 mmol) and *N*-(2-aminoethyl)-4-methylbenzenesulfonamide (synthesized according to previous report) [1] (0.6 g, 2.8 mmol) were dissolved in 200 mL of chloroform and kept with stirring at 35 °C for 48 h. The reaction mixture was washed using water (3 x 100 mL) to remove the excess amine and the organic layer was collected. It was dried over sodium sulphate and the solvent was removed to get pure compound, **PM-ER-OMe** (0.232 g, 32%). It was characterized by ^1H, ^{13}C NMR and HRMS.

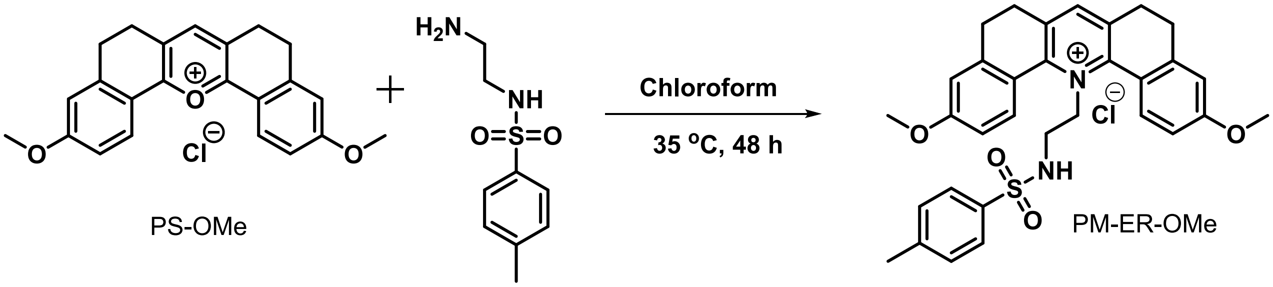

Yield: 32%

^{1}H NMR (500 MHz, $CDCl_3$): 7.9 (s, 1H), 7.79 (d, J = 10 Hz, 2H), 7.41 (d, J = 5 Hz, 2H), 7.04 (d, J = 10 Hz, 2H), 6.92 (m, 2H), 6.85 (d, J = 5 Hz, 2H), 5.35 (t, J = 5 Hz, 2H), 3.81 (s, 6H), 3.36 (m, 2H), 3.03 (d, J = 15 Hz, 2H), 2.76 (d, J = 10 Hz, 2H), 2.62 (m, 4H), 2.22 (s, 3H).

^{13}C NMR (125 MHz, $CDCl_3$): 162.8, 153.7, 144.6, 143.4, 142.8, 136.6, 136.1, 130.3, 129.7, 126.7, 120.9, 114.5, 113.6, 63.9, 55.7, 43.7, 28.2, 21.4

HRMS: Calculated mass = 541.2156, Experimental mass = 541.21676

Synthesis of PM-ER-OH:

PM-ER-OMe (0.1 g, 0.17 mmol) was dissolved in dry dichloromethane and cooled to -5 °C keeping the round-bottom flask in ice and salt mixture. After 30 minutes, under inert condition, BBr_3 in dichloromethane (1.5 mL, 6 mmol) was added dropwise. Then it was kept under stirring for overnight at 35 °C. After confirming the completion of the reaction, excess of BBr_3 was quenched by adding distilled water slowly. Finally, the precipitate was collected by filtration and it was washed several times with distilled water to get **PM-ER-OH** (0.08 g, 86%). It was characterized by ^{1}H, ^{13}C NMR and HRMS.

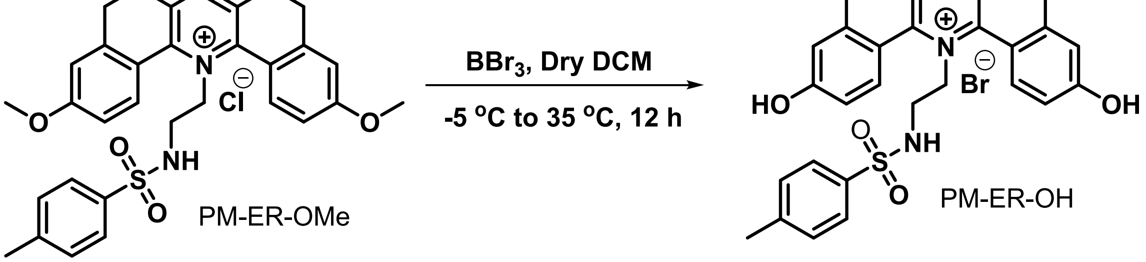

^{1}H NMR (500 MHz, CD_3OD): 8.01 (s, 1H), 7.86 (d, *J* = 5 Hz, 2H), 7.31 (d, *J* = 5 Hz, 2H), 7.15 (d, *J* = 10 Hz, 2H), 6.82 (m, 4H), 5.22 (t, *J* = 5 Hz, 2H), 3.04 (m, 4H), 2.72 (m, 4H), 2.56 (t, *J* = 5 Hz, 2H), 2.25 (s, 3H).

^{13}C NMR (125 MHz, CD_3OD): 161.5, 153.4, 144.8,143.6, 141.8, 136.6, 136.3, 130.3, 129.4, 126.2, 119.9, 115.1, 114.6, 62.8, 42.1, 27.8, 27.7, 19.9

HRMS: Calculated mass = 513.1843, Experimental mass = 513.18591

2. Optical Measurements

Electronic absorption spectra were recorded in a Shimadzu UV-2600 UV-Vis spectrophotometer and emission studies were performed in Horiba Fluorolog ─3 Jovin Yoon. The stock solution of all the molecules were prepared in spectroscopy grade DMSO and diluted with phosphate buffer as per need. The pH solutions were made by using phosphate buffer and adjusted by using dilute HCl/ NaOH solution.

2.1 Basic photophysical data of the molecules

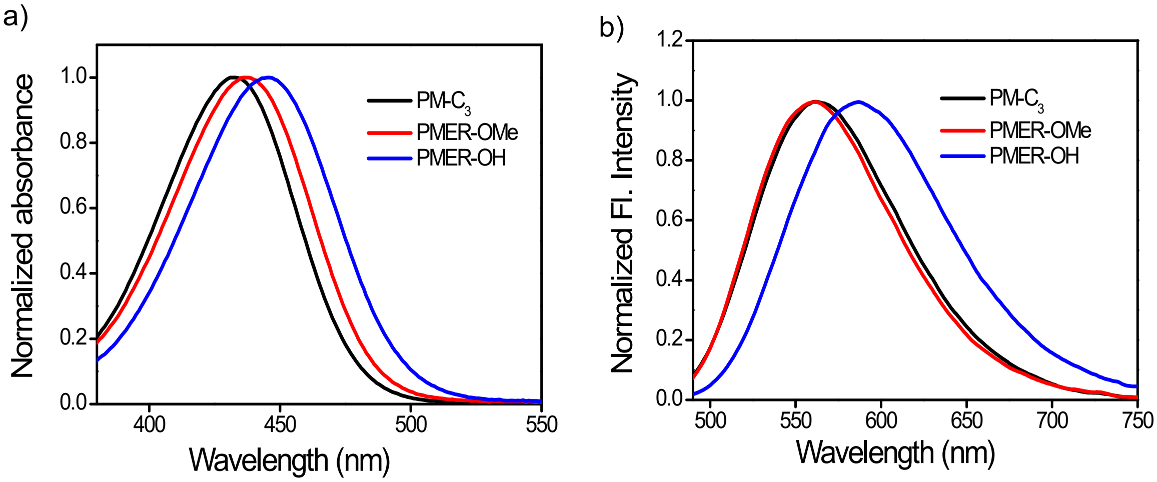

Fig S2: a) Normalized absorbance and b) Normalized emission spectra of **PM-C_3**, **PM-ER-OMe** and **PM-ER-OH** (10 μM for all) in chloroform.

2.2 PM-C_3 and PM-ER-OMe pH response:

To understand whether the fluorescence change of **PM-ER-OH** with pH variation is due to the two –OH groups present in it, we studied the effect of pH on our control molecules, i.e., **PM-C_3** and **PM-ER-OMe**. In the case of **PM-C_3**, we could not find any significant change in the peak maximum and intensity for both absorbance and emission with change in pH from 4 to 10. However, in the case of **PM-ER-OMe**, though there was no shift in peak maximum with increase in pH, the intensity decreased for both absorbance and emission. As the fluorophore core is same for both molecules, the decrease in intensity of the peak for **PM-ER-OMe** may be due to the weak photo induced electron transfer (PET) effect coming from the sulphonamide group at the basic pH value. But for both molecules, there was no shift in the peak maximum in the emission spectra.

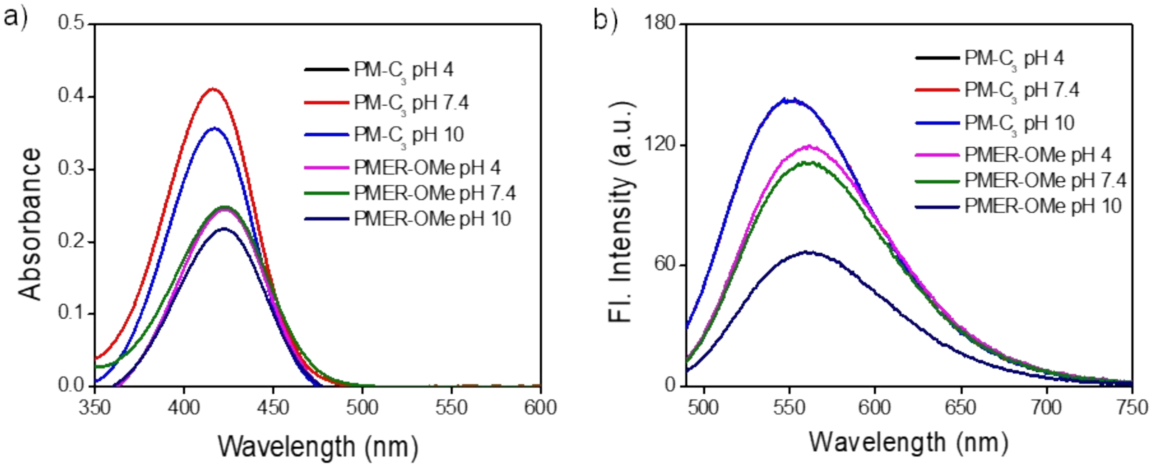

Fig S3: a) absorbance and b) emission spectra of PM-C_3 and PM-ER-OMe in PBS buffer of pH 4, 7.4 and 10.

2.3 Fluorescence Quantum Yield measurement:

Fluorescence quantum yields of the molecules were determined using Coumarin 153 as the Φ_f reference according to the literature method.

Quantum yields were determined following this equation:

$\Phi_s=\Phi_f$ x (A_r/A_f) x (F_s/F_r)

Where ‘s’ and ‘r’ represent the synthesized and reference samples, respectively. A, Φ_f, and F were the absorbance (≤ 0.07) at λ_{ex}, refractive index of the used spectroscopy grade solvent, and the integrated area under the corrected emission spectrum, respectively.

3. Laser wavelength compatibility:

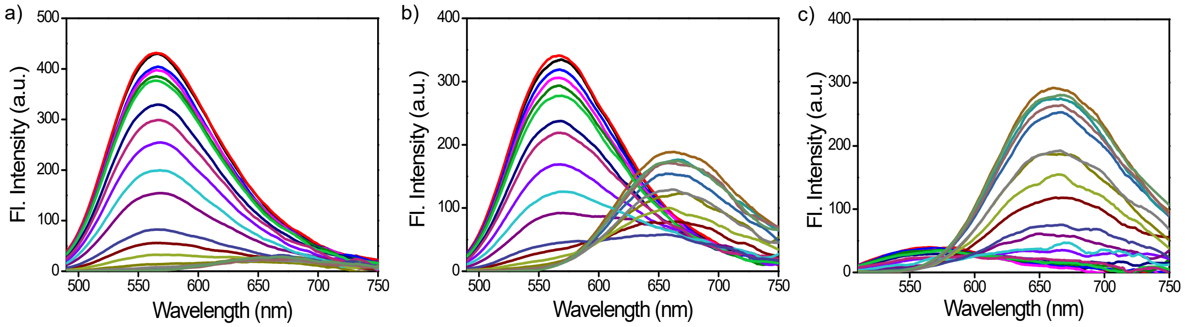

Fig S4: a) Emission Spectra of PM-ER-OH from pH 4 - 10 (λ_{ex} = 405 nm). b) Fluorescence response of the probe at pH 4 - 10 when excitation wavelength is shifted to 458 nm. c) Emission spectra of PM-ER-OH when the excitation wavelength is shifted to 488 nm at pH 4 - 10.

4. Selectivity of PM-ER-OH

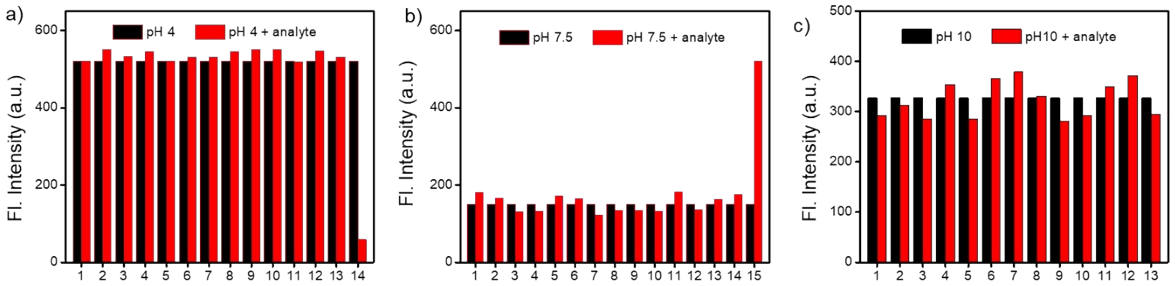

Fig S5: a) Selectivity test was performed where at pH 4, different biologically relevant analytes (1. K^+, 2. Fe^{2+},3. Na^+, 4. Ca^{2+}, 5. Mg^{2+},6. Al^{3+},7. Zn^{2+},8. AcO^-, 9. HOCl, 10. H_2S, 11. H_2O_2, 12. GSH, 13. O^{2-} , 14. OH^-, 15. H^+) were added with PM-ER-OH (10 μM) And a bar diagram was made taking the intensity maximum of the emission spectra. b) & c) similar selectivity test was done at pH 7.5 and 10 and bar diagram was constructed.

5. General Strategies for cellular studies:

5.1 Cell culture: The HeLa cells, that were obtained from American Type Culture Collection (ATCC, Manassas, VA), were grown in Dulbecco's Modified Eagle's medium (DMEM) supplemented with 10% Fetal Bovine Serum (FBS) and 1% penicillin-streptomycin (PS) antibiotics at 37 ℃ in humidified environment of 5% CO_2. Cells were plated on 6-well plates, and allowed to adhere for 12 h.

5.2 MTT assay: HeLa cells were seeded in three 96-well plates (1 × 10^4 cells per well) and allowed to adhere for 12 h. The following day, different concentrations of **PM-ER-OMe**, **PM-ER-OH**, and **PM-C$_3$** (0, 2.5, 5, 10, 25, 50, and 100 μM) were added to the wells and incubated for 24 h. Thereafter, MTT solution (5 mg/mL, 10 μL per well) was added mixed with DMEM. Following 4 h incubation, the MTT solution was replaced with 100 μL of DMSO and absorbance of the formed formazan crystals was measured at 570 nm. The percentage of cellular viability was calculated according to the following equation:

$$Cell\ viability\ (\%) = \frac{OD\ of\ treated\ cells}{OD\ of\ control} \times 100\ (\%)$$

5.3 Co-localization: For the colocalization experiment, the HeLa cells (1 × 10^4 cells) were seeded in μ-Dish 35 mm microscopy dishes. 24 h after adherence, media were replaced with 10 μM **PM-ER-OH** and 10 μM **PM-C$_3$**, and allowed to incubate for 20 min in PBS at 37 ℃. Thereafter, the cells were washed three times with PBS, and replaced with PBS containing 1 μM ER tracker solution. 20 minutes later, the cells were washed with PBS three times and imaged under Zeiss LSM710 CLSM. The samples were excited with 405 nm and 488 nm lasers. Image analysis was performed using Image j.

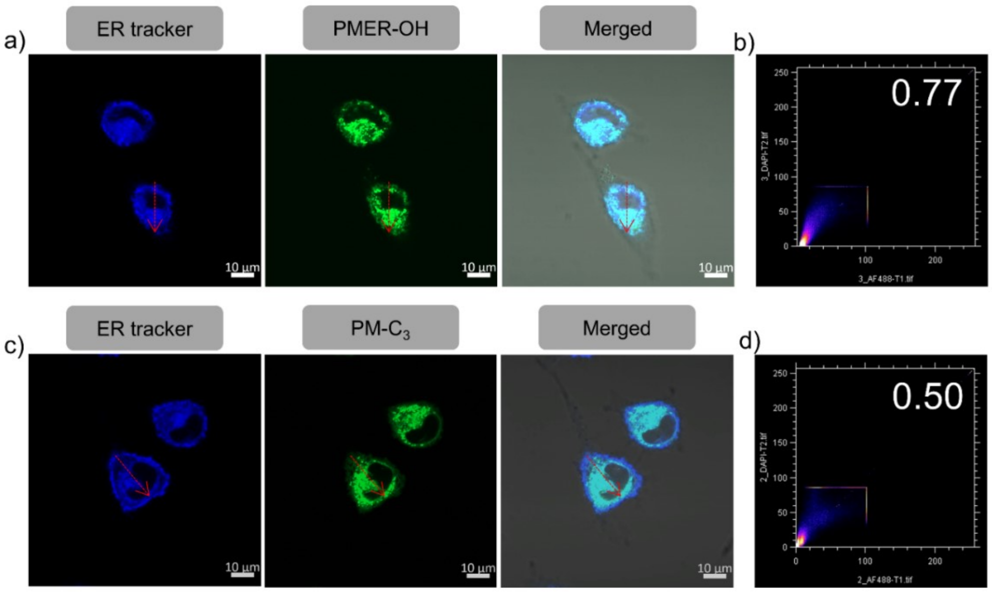

Fig S6: Colocalization analysis. a) CLSM images of ER tracker and **PM-ER-OH** incubated live HeLa cells in blue, green and merged channels. b) Correlation of **PM-ER-OH** and ER tracker intensities. c) CLSM images of ER tracker and **PM-C_3** stained live HeLa cells in blue, green and merged channels. d) Correlation of **PM-C_3** and ER tracker intensities. Pearson's coefficients calculated using ImageJ are mentioned in the inset. Scale bar 10 µm.

5.4 Photobleaching/photostability Studies: HeLa cells (1×10^4 cells) were seeded in µ-Dish 35 mm microscopy dishes. 24 h after adherence, media was replaced with 10 µM **PM-ER-OH** and allowed to incubate for 20 min in PBS at 37 ℃. Thereafter, the cells were washed three times with PBS, and replaced with PBS containing 1 µM ER tracker solution. 20 minutes later, the cells were washed with PBS three times and imaged under Zeiss LSM710 CLSM. For photostability, within 600 s, 10 scans with high intensity laser excitation were acquired at 405 nm for both the dyes.

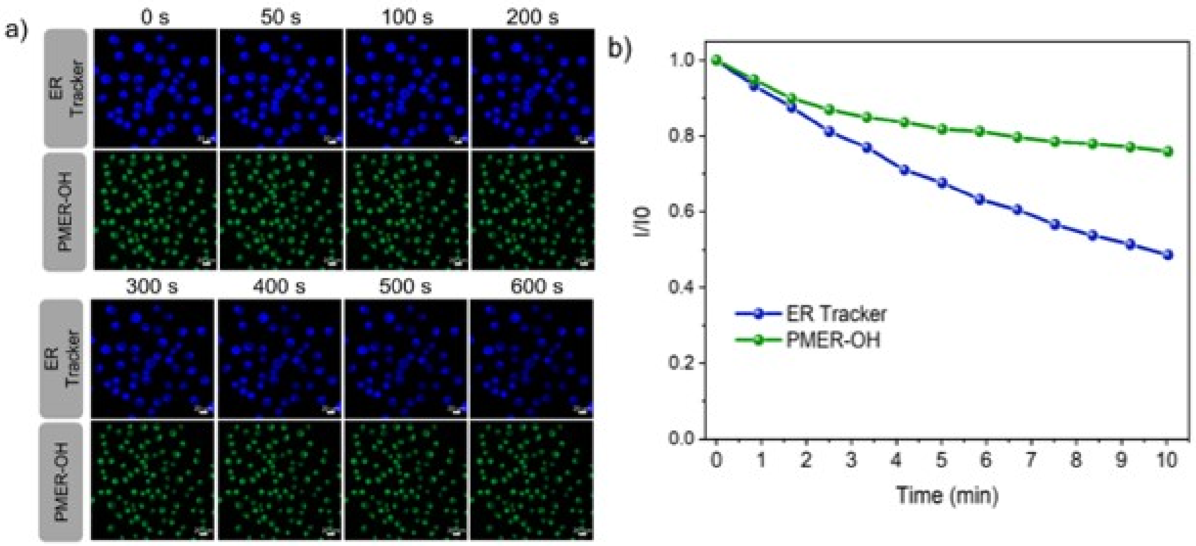

Fig S7: Comparison of photostability of **PM-ER-OH** with commercially available ER tracker. (a) HeLa cells were treated with 1 µM of ER-Tracker Blue-White DPX dye and 10 µM of **PM-ER-OH** separately. Then cells were kept under light irradiation for 10 mins with intervals of 10 s. Images were acquired every 60 s. Scale bar is 20 µm. (b) The fluorescence intensity was measured from the cells and a comparison graph for the intensity ratio at each time after photobleaching was plotted. Scale bar 20 µm.

5.5 pH variation: HeLa cells were incubated at 37 ℃ for 30 min in high K^+ buffer (30 mM NaCl, 120 mM KCl, 1 mM $CaCl_2$, 0.5 mM $MgSO_4$, 1mM Na_2HPO_4, 5 mM glucose, 20 mM sodium acetate and 20 mM MES) of various pH values (pH 4.2 – 9.5). Next 25 µL (10 µM), a H+ /K+ antiporter nigericin was added and incubated for 20 min to equilibrate the intracellular pH with the pH buffer. The cells were then incubated with 20 µL of the **PM-ER-OH** (20 µM) for 20 min, washed with PBS and observed under CLSM under excitation at 405 nm, 458 nm, and 488 nm. Image processing was performed with Zeiss software (Zen 3.2) and Image analysis was performed with Image J.

5.6 Heat shock imaging: HeLa cells were seeded in 6-well plates and incubated at 37 ℃ for 24 h. The cells were divided into two sets: (i) normal heating to 41 ℃ and 45 ℃, and (ii) pre heated at 40 ℃ for 3 h, and subsequent heating to 41 ℃ and 45 ℃. For the (i) normal heating set, the cells were kept at 41 °C and 45 °C for 30 min each with **PM-ER-OH**. For (ii) pre-heating, the cells were kept at 40 ℃ for 3 h, and later incubated with **PM-ER-OH** at 41 ℃ and 45 ℃ for 30 min. Both

sets were finally imaged under CLSM in two channels (green and red) using three different excitation sources i.e., 405 nm, 458 nm, and 488 nm.

6. ^{1}H, ^{13}C NMR and HRMS of PM-C_3, PM-ER-OMe and PM-ER-OH:

^{1}H NMR of PM-C_3

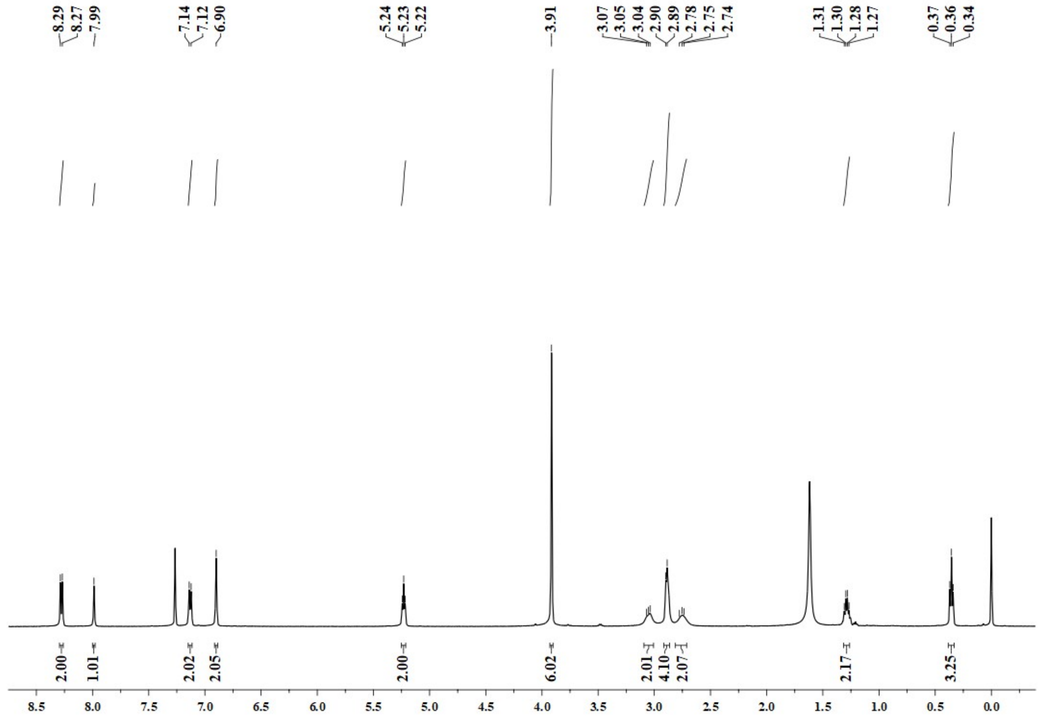

^{13}C NMR of PM-C_3

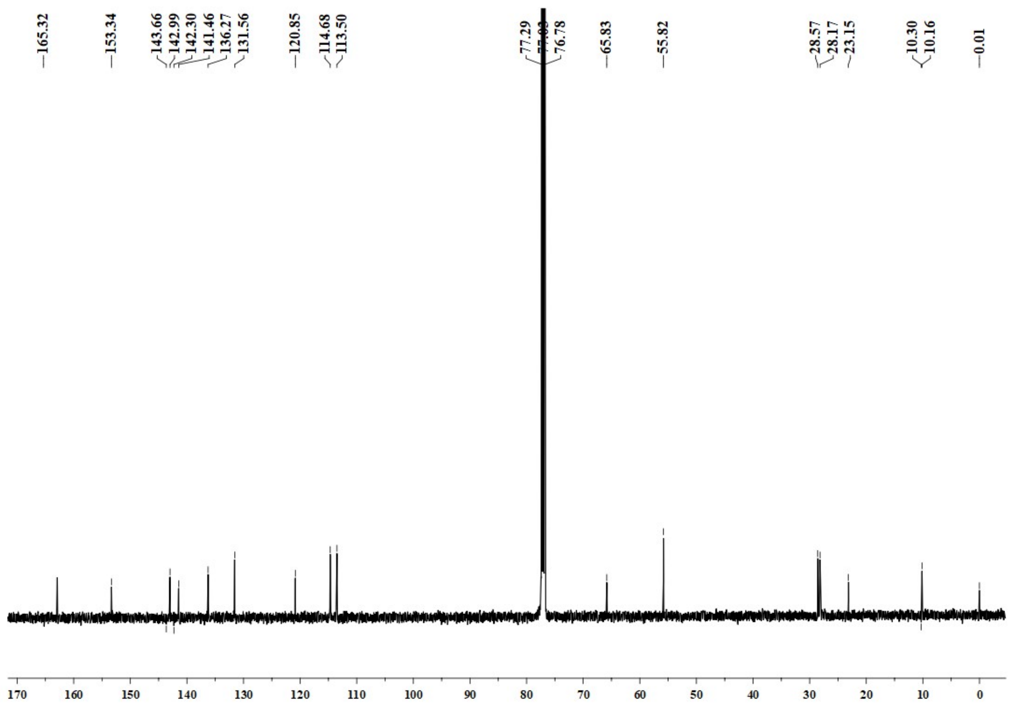

^{1}H NMR of PM-ER-OMe

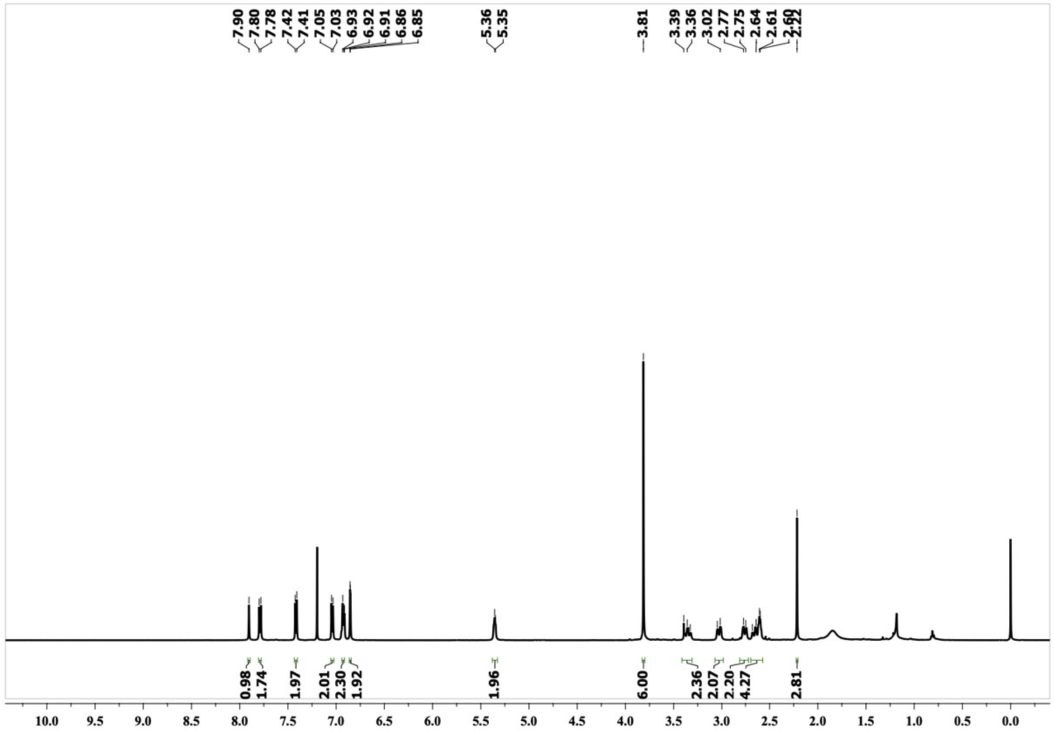

^{13}C NMR of PM-ER-OMe

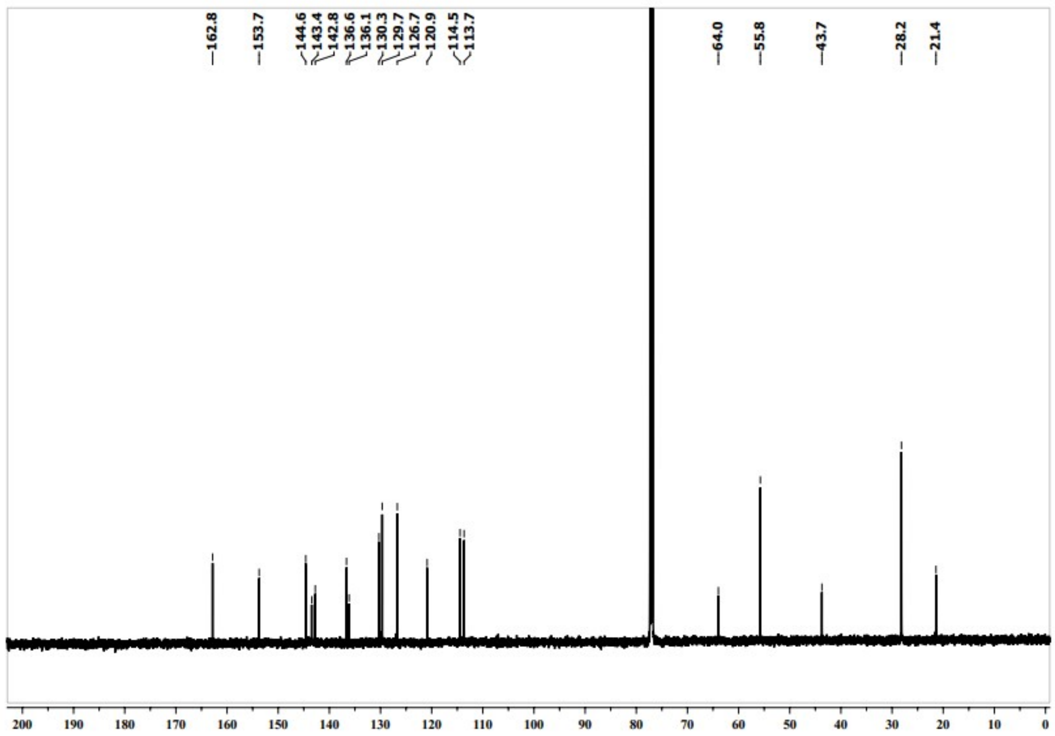

^{1}H NMR of PM-ER-OH

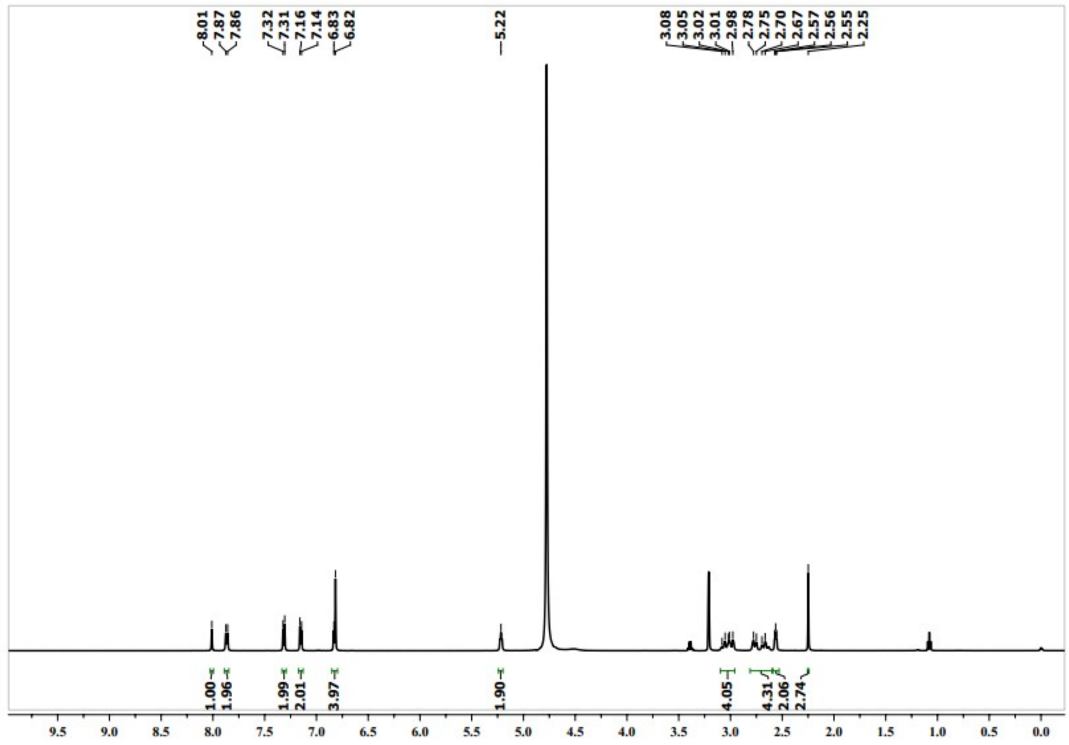

^{13}C NMR of PM-ER-OH

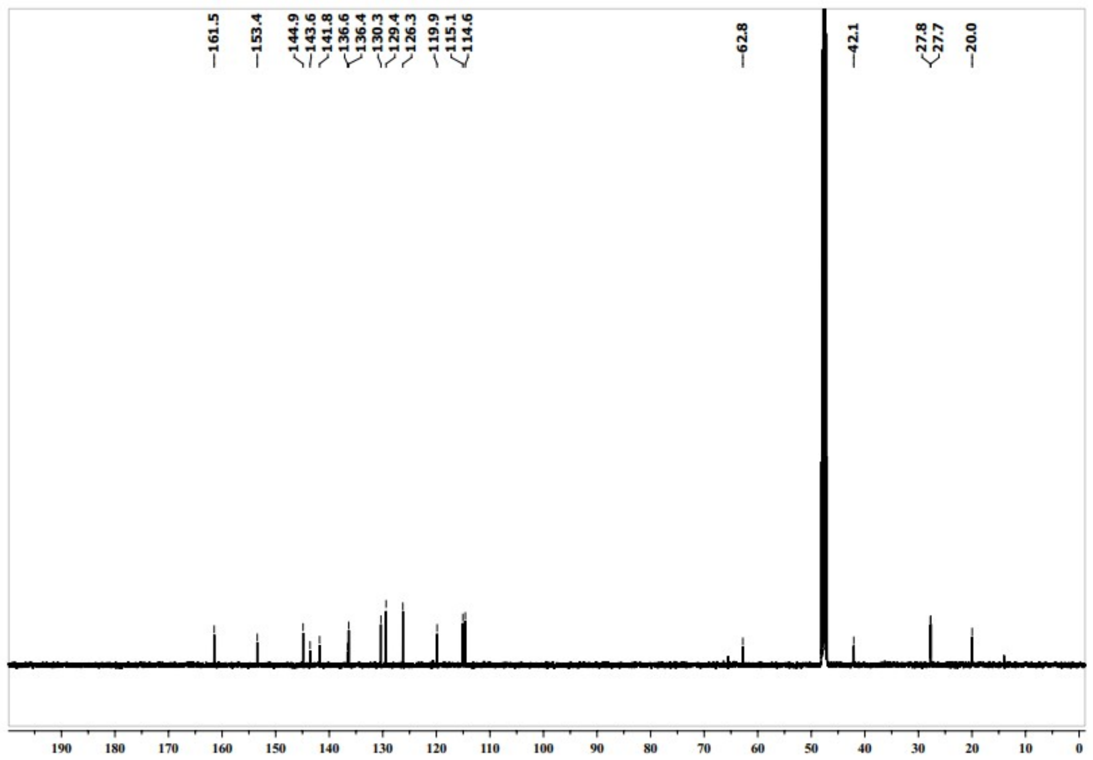

HRMS of PM-C_3

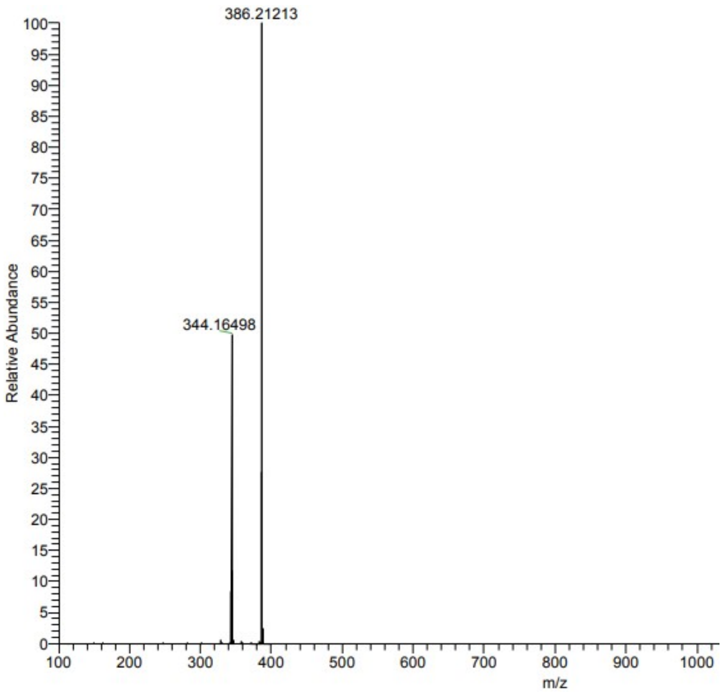

HRMS of PM-ER-OMe

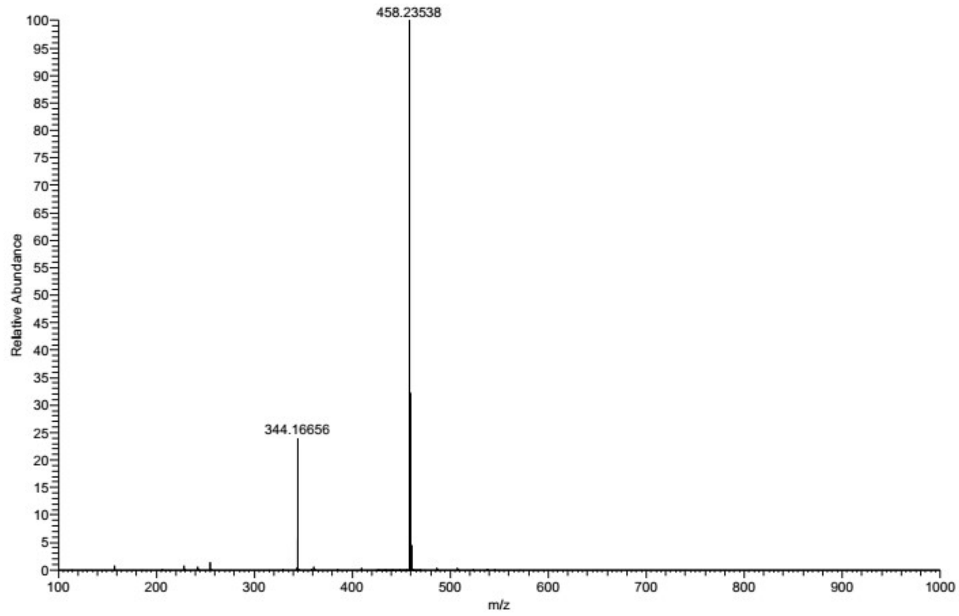

HRMS of PM-ER-OH

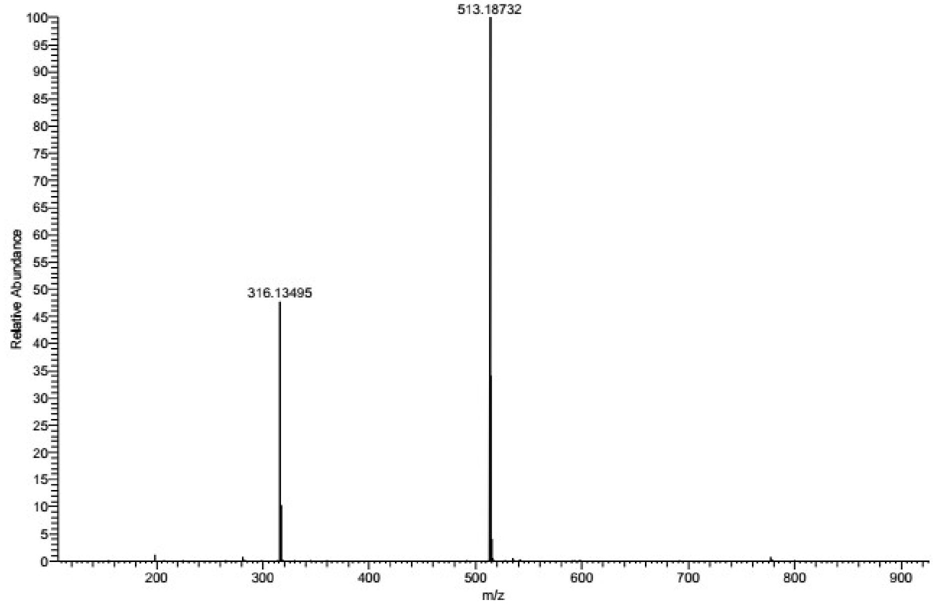

References:

1. Li, S.; Zhou, D.; Li, Y.; Liu, H.; Wu, P.; Ou-Yang, J.; Jiang, W. and Li, C. Efficient Two-Photon Fluorescent Probe for Imaging of Nitric Oxide during Endoplasmic Reticulum Stress. *ACS Sens.*, **2018**, 3, 2311−2319
